# Competitive binding at a nicotinic receptor transmembrane site of two α7-selective positive allosteric modulators with differing effects on agonist-evoked desensitization

**DOI:** 10.1016/j.neuropharm.2011.07.035

**Published:** 2011-12

**Authors:** Toby Collins, Gareth T. Young, Neil S. Millar

**Affiliations:** Department of Neuroscience, Physiology & Pharmacology, University College London, Gower Street, London, WC1E 6BT, United Kingdom

**Keywords:** Acetylcholine receptor, Ligand-gated ion channel, Nicotinic receptor, Positive allosteric modulator

## Abstract

Positive allosteric modulators (PAMs) of nicotinic acetylcholine receptors (nAChRs) have attracted considerable interest as a novel area of therapeutic drug discovery. Two types of α7-selective PAMs have been identified (type I and type II). Whilst both potentiate peak agonist-induced responses, they have different effects on the rate of agonist-induced receptor desensitization. Type I PAMs have little or no effect on the rapid rate of desensitization that is characteristic of α7 nAChRs, whereas type II PAMs cause dramatic slowing of receptor desensitization. Previously, we have obtained evidence indicating that PNU-120596, a type II PAM, causes potentiation by interacting with an allosteric transmembrane site. In contrast, other studies have demonstrated the importance of the ‘M2–M3 segment’ in modulating the effects of the type I PAM NS1738 and have led to the proposal that NS1738 may interact with the extracellular N-terminal domain. Here, our aim has been to compare the mechanism of allosteric potentiation of α7 nAChRs by NS1738 and PNU-120596. Functional characterization of a series of mutated α7 nAChRs indicates that mutation of amino acids within a proposed intrasubunit transmembrane cavity have a broadly similar effect on these two PAMs. In addition, we have employed a functional assay designed to examine the ability of ligands to act competitively at either the orthosteric or allosteric binding site of α7 nAChRs. These data, together with computer docking simulations, lead us to conclude that both the type I PAM NS1738 and the type II PAM PNU-120596 bind competitively at a mutually exclusive intrasubunit transmembrane site.

## Highlights

► We have examined two positive allosteric modulators of the α7 nicotinic receptor. ► The two modulators have differing effects on agonist-evoked desensitization. ► We provide evidence that they act at a common transmembrane binding site.

## 1 Introduction

Nicotinic acetylcholine receptors (nAChRs) are a diverse family of pentameric neurotransmitter receptors (Millar and Gotti, 2009) and are members of the larger super-family of ligand-gated ion channels (Lester et al., 2004). Although many nAChR subtypes have been identified in the mammalian brain, α7 nAChRs are somewhat unusual in forming functional homomeric receptors (Couturier et al., 1990). In recent years, the α7 nAChR has attracted considerable interest as a target for drug discovery (Haydar and Dunlop, 2010) due, in part, to evidence that it may have a cognitive enhancing role. Nicotinic receptors are complex allosteric proteins with the possibility of a variety of modulatory ligand binding sites (Taly et al., 2009). In addition to α7-selective agonists and antagonists, a number of α7-selective positive allosteric modulators (PAMs) have been reported in recent years (for recent reviews, see Faghih et al., 2008; Malysz et al., 2009). Recent studies have also provided evidence that α7-selective PAMs may have cognitive enhancing effects and, as a consequence, may be of use in the treatment of neurological and psychiatric disorders such as Alzheimer’s disease and schizophrenia (Timmermann et al., 2007). Positive allosteric modulators have been classified as either ‘type I’ or ‘type II’ potentiators, on the basis of a difference in their effect on desensitization (Bertrand and Gopalakrishnan, 2007). Whilst both types of PAM enhance agonist-evoked responses, type II PAMs cause a dramatic reduction in receptor desensitization (Grønlien et al., 2007; Hurst et al., 2005).

Two α7-selective PAMs that have been studied in some detail are NS1738, a type I PAM (Timmermann et al., 2007) and PNU-120596, a type II PAM (Hurst et al., 2005). Recent evidence has suggested that the allosteric binding site for PNU-120596 is located in an intrasubunit cavity located between the four transmembrane (TM) helices (TM1–TM4; also referred to as M1–M4) of the α7 subunit (Young et al., 2008). This site is similar to the allosteric site that has been proposed for modulators such as anaesthetics and neurosteroids acting on GABA- and glycine-gated ion channels (Hosie et al., 2006; Mihic et al., 1997; Ye et al., 1998). Further evidence for modulatory binding sites for anaesthetics being located in the transmembrane region has come from photoaffinity labelling studies conducted with purified *Torpedo* nAChRs (Garcia et al., 2007; Ziebell et al., 2004). In addition, there is evidence to indicate that the type I PAM ivermectin, a large macrocyclic lactone, interacts with a transmembrane site on nAChRs (Collins and Millar, 2010; Sattelle et al., 2009).

Due to the profound differences in their effects on receptor desensitization, it is possible that α7-selective type I and type II PAMs may interact at distinct allosteric sites. Indeed, recent studies with NS1738 demonstrate that a 10 amino acid region described as being the ‘M2–M3 segment’ has a selective effect on modulation by the type I PAM NS1738 and have led to the suggestion that NS1738 may interact with the N-terminal extracellular domain of α7 nAChRs (Bertrand et al., 2008). Here, we have examined the allosteric modulation of α7 nAChRs by a type I PAM (NS1738) and a type II PAM (PNU-120596). We have examined the effects on allosteric modulation of a series of site-directed mutations and have performed computer docking simulations with an α7 nAChR homology model. In addition, we have employed an assay designed to examine whether type I and type II PAMs bind competitively. Based on these studies, our conclusion is that NS1738 and PNU-120596 bind competitively at a shared allosteric transmembrane site.

## 2 Materials and methods

### 2.1 Materials

NS1738 (Timmermann et al., 2007) was generously provided by Daniel Timmermann (NeuroSearch, Denmark). Methyllycaconitine (MLA) and PNU-120596 (Hurst et al., 2005) were obtained from Tocris Bioscience (Bristol, UK). All other chemicals were obtained from Sigma (Poole, UK).

### 2.2 Subunit cDNAs and plasmid expression vectors

All experiments were performed with human α7 nAChRs with the exception of experiments that directly compared mutated and wild type receptors, which were performed with rat α7 nAChRs. Human nAChR α7 subunit cDNA in plasmid pSP64GL and rat nAChR α7 subunit cDNA in pcDAN1neo have been described previously (Broadbent et al., 2006; Séguéla et al., 1993). Mutated rat α7 cDNAs have been described previously and were used (rather than mutated human cDNAs) to enable a direct comparison with data reported previously with PNU-120596 (Young et al., 2008).

### 2.3 Xenopus oocyte electrophysiology

*Xenopus laevis* oocytes were isolated and defolliculated as described previously (Young et al., 2007). To express rat α7 nAChR (for experiments comparing mutant and wild type receptors), oocyte nuclei were injected with cDNAs cloned downstream from a CMV promoter in plasmid expression vectors (Young et al., 2007). To express human α7 nAChRs (for experiments other than those examining point mutations) *in vitro* transcribed cRNA was injected into the oocyte cytoplasm. *In vitro* transcription of cRNA was carried out using mMESSAGE mMACHINE T7 transcription kit (Ambion, Huntington, UK). Oocytes were injected with 2–20 ng DNA or 2 ng cRNA per oocyte in a volume of 18.4 nl using a Drummond variable volume microinjector. Experiments were performed, typically, 2–4 days after injection of oocytes with cDNA or cRNA. Two-electrode voltage-clamp recordings were performed (with the oocyte membrane potential held at −60 mV), as described previously (Young et al., 2007) using either an Axon Geneclamp 500B amplifier, Axon DigiData 1200 and pClamp software (Molecular Devices, Sunnyvale, CA) or with a Warner Instruments OC-725C amplifier (Harvard Apparatus, Edenbridge, UK), PowerLab 8SP and Chart 5 software (AD Instruments, Oxford, UK). Agonists and PAMs were applied to oocytes using a BPS-8 solenoid valve solution exchange system (ALA Scientific Inc., Westbury, NY), controlled by either pClamp or Chart software. For most experiments, acetylcholine was used at either a maximal or EC_20_ concentration (1 mM and 50 μM, respectively) and PAMs were used at maximal concentrations (10 μM for NS1738 and 3 μM for PNU-120596). Except were stated otherwise, PAMs were pre-applied for 20 s and were then co-applied with acetylcholine.

### 2.4 Computer docking simulations

Computational molecular docking of NS1738 were performed with AutoDock 4 (Morris et al., 1998) using a homology model of the human α7 nAChR transmembrane region, as described previously (Cheng et al., 2007; Young et al., 2008). To avoid bias, initial studies employed a ‘blind docking’ approach (Hetényi and van der Spoel, 2006) in which no assumptions were made concerning where within the transmembrane region ligands might be expected to bind. Flexibility of rotatable bonds in docked ligands was permitted during the docking simulation but with a rigid homology model. Subsequently, docking simulations were performed in which flexibility was allowed for the side chains of five amino acid [S222 (TM1), A225 (TM1), M253 (TM2), F455 (TM4) and C459 (TM4)] that have been implicated by site-directed mutagenesis as influencing potency of allosteric modulators. Predicted Gibbs free energy of binding (Δ*G*) was calculated as described (Huey et al., 2007; Morris et al., 1998), as was the predicted equilibrium constant for binding (*K*_eq_), using the equation *K*_eq_ = *e*^–Δ*G*/*RT*^ (where *R* = gas constant, *T* = absolute temperature).

## 3 Results

### 3.1 Characterization of mutated α7 nAChRs

**Fig. 1:**
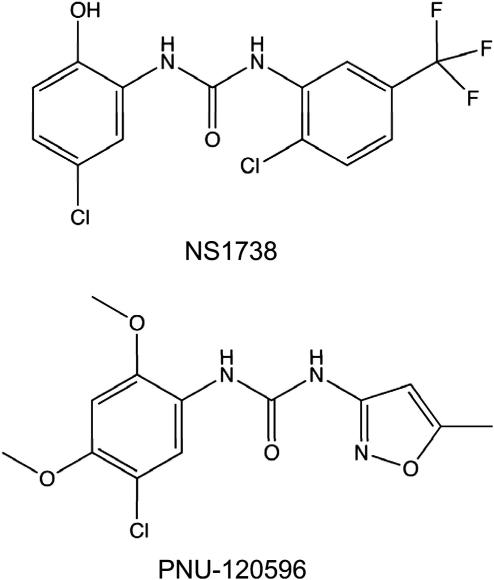
Chemical structure of α7-selective potentiators NS1738 and PNU-120596. Both NS1738 (a type I PAM) and PNU-120596 (a type II PAM) are urea compounds containing a halogenated aromatic group.

**Fig. 2:**
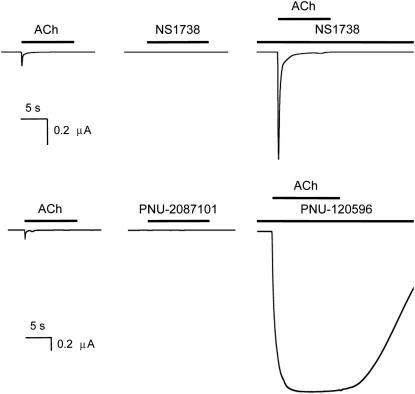
Positive allosteric modulation of α7 nAChRs by NS1738 and PNU-120596. Representative traces are shown illustrating agonist-evoked responses with α7 nAChRs expressed in *Xenopus* oocytes. Application of acetylcholine (1 mM) resulted in rapidly desensitizing responses that are typical of α7 nAChRs (left hand traces). Pre-application (20 s) followed by co-application of either NS1738 (10 μM) or PNU-120596 (3 μM) resulted in potentiation of responses evoked by acetylcholine (right panels). In contrast, no agonist activity was observed when NS1738 (10 μM) and PNU-120596 (3 μM) were applied in the absence of acetylcholine (middle traces). Applications of acetylcholine and PAMs are indicated by horizontal lines.

The ability of NS1738, a type I PAM, and PNU-120596, a type II PAM, (Fig. 1) to act as allosteric modulators of α7 nAChRs was examined by two-electrode voltage-clamp recording in *Xenopus* oocytes. Neither compound had significant agonist activity on α7 nAChRs at the concentrations tested (Fig. 2A and B) but, when co-applied with acetylcholine, both caused substantial potentiation of acetylcholine-evoked responses (Fig. 2A and B). The representative traces shown in Fig. 2 illustrate an important difference between type I and type II PAMs. NS1738 has little or no effect on the rapid desensitization of α7 nAChRs, whereas PNU-120596 causes a dramatic slowing of receptor desensitization.

We have previously reported studies indicating that PNU-120596 interacts with an intrasubunit cavity in α7 nAChRs (Young et al., 2008), whereas other recent studies have provided evidence that potentiation by NS1738 is influenced by the α7 ‘M2–M3 segment’ and, leading to the suggestion that it may interact with the extracellular N-terminal domain (Bertrand et al., 2008). With the aim of exploring in greater detail the interaction of these allosteric modulators with the α7 nAChR, the influence of 11 point mutations was examined (Table 1). All of these mutations are predicted to lie within a cavity located between the four α helices of the α7 subunit and their effects on potentiation by PNU-120596 have been reported previously (Young et al., 2008).

**Fig. 3:**
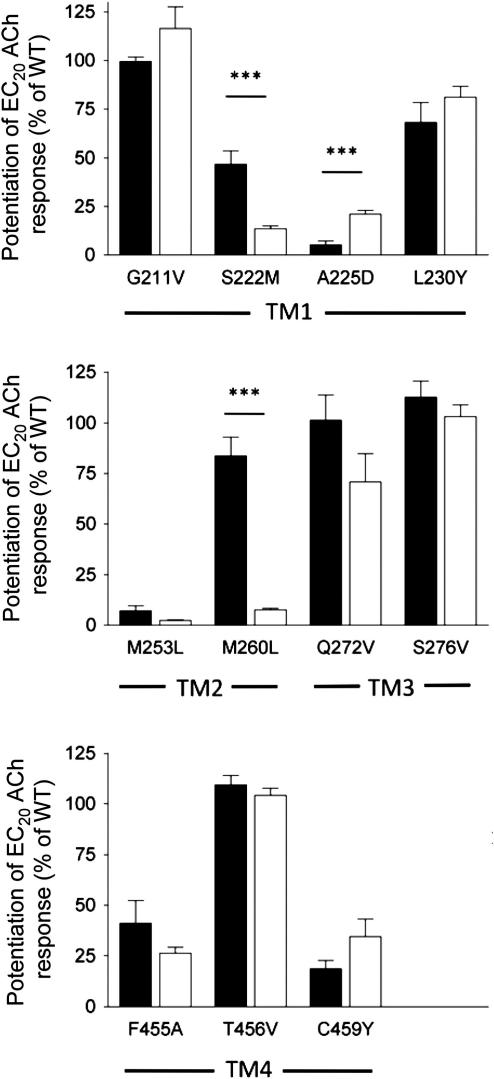
Influence of α7 nAChR transmembrane point mutations upon potentiation by NS1738 and PNU-120596. Histograms illustrating the influence of α7 point mutations on the level of potentiation caused by maximal concentrations of NS1738 (10 μM; open bars) and PNU-120596 (3 μM; filled bars). Potentiation of agonist-evoked responses were determined with an EC_20_ concentration of acetylcholine (see Table 1). Data are means ± SEM of 3–10 independent experiments (***P < 0.001; Student’s *t*-test). Data for PNU-120596 are taken from (Young et al., 2008).

**Fig. 4:**
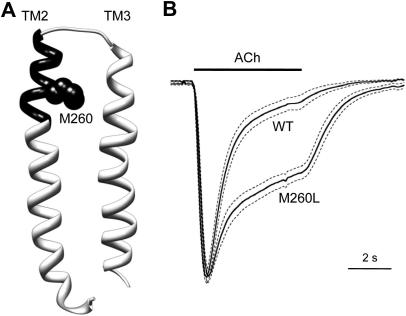
Altered receptor desensitization in α7 nAChRs containing the M260L mutation. A) Model of the TM2 and TM3 transmembrane region derived from the α7 nAChR homology model (Cheng et al., 2007). The location of a 10 amino acid region ‘M2-M3 segment’ (AEIMPATSDS) described by Bertrand et al. (2008) is shaded in black. Also illustrated (as black spheres) is the side chain of M260, the amino acid that when mutated has a selective effect on NS1738. B) Differences in desensitization of wild type α7 and α7^M260L^ nAChRs are illustrated by responses to 5 s applications of an EC_20_ concentration of acetylcholine. Solid lines represent normalized means of 9 (wild type α7) or 10 (α7^M260L^) independent recordings. Dotted lines represent SEMs.

On wild type α7 nAChRs, NS1738 potentiated responses to an EC_20_ concentration of acetylcholine (50 μM) by 39.5 ± 2.7 fold with an EC_50_ value of 6.7 ± 1.8 μM. Similarly, previous studies from our laboratory (Young et al., 2008) found that, on wild type α7 nAChRs, PNU-120596 potentiated responses to an EC_20_ concentration of acetylcholine by 36.7 ± 4.1 fold with an EC_50_ value of 1.5 ± 0.2 μM. The ability of NS1738 to potentiate agonist-evoked responses was also examined on 11 mutated α7 nAChRs. In order to facilitate a comparison of the relative effects of the mutations on NS1738 with those determined previously for PNU-120596, the level of potentiation has been expressed as a percentage of that observed with wild type α7 receptors (Fig. 3). Absolute levels of potentiation observed with NS1738 on mutant α7 nAChRs are presented in Table 1. Overall, the effect of transmembrane mutations on potentiation by NS1738 and PNU-120596 was broadly similar, suggesting that these PAMs may interact via a similar site on α7 nAChRs. However, some differences are apparent (Fig. 3). Two mutations (S222M and A225D), both of which are located in TM1, significantly reduced levels of potentiation by NS1738 and PNU-120596 but did so to different extents. S222M had a significantly greater effect (*P* < 0.001) on NS1738, whereas A225D had a significantly greater effect (*P* < 0.001) on PNU-120596 (Fig. 3). One mutation M260L, located in TM2 did not have a significant effect on PNU-120596 but caused a dramatic reduction in potentiation by NS1738 (Fig. 3). A notable feature of α7 nAChRs containing the M260L mutation is an alteration in the desensitization profile caused by prolonged agonist exposure (Fig. 4B). Whereas high concentrations of acetylcholine causes rapid and complete receptor desensitization in wild type α7 nAChRs, an initial rapid phase of desensitization was observed which was followed by a period of sustained receptor activation in the M260L mutant (Fig. 4B). The extent of receptor desensitization was determined in response to application of an EC_20_ concentration of acetylcholine. The time for responses to decay to half of the peak response level during continued agonist application was significantly slower for M260L α7 nAChRs (302 ± 45 ms, *n* = 10) than for wild type (87 ± 10 ms, *n* = 9; *P* = 0.001). The total charge transfer (area under the curve) during a 5 s application of acetylcholine (EC_20_) was also calculated and was significantly greater (2.0 ± 0.1 fold, *n* = 10, *P* < 0.001) for M260L than for wild type α7 nAChRs.

### 3.2 Type II PAM-induced recovery from desensitization

**Fig. 5:**
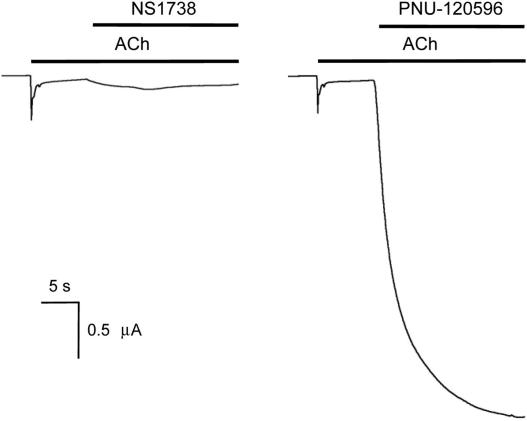
PNU-120596 (a type II PAM) facilitates recovery of α7 nAChRs from desensitization. Exposure of α7 nAChR to a high concentration of acetylcholine (1 mM) resulted in rapid desensitization. The subsequent co-application of NS1738 (10 μM) to desensitized α7 nAChRs had little effect (left hand trace). In contrast, the co-application of PNU-120596 after desensitization by acetylcholine (3 μM) causes rapid opening, indicating a recovery from the receptor’s desensitized state (right hand trace).

As is illustrated in Fig. 2, NS1738 and PNU-120596 differ markedly in their effects on receptor desensitization. A further striking difference between type I and type II PAMs is their ability to facilitate recovery of α7 nAChRs from desensitization (Young et al., 2008). After desensitization of α7 nAChRs by prolonged exposure to a high concentration of acetylcholine, the subsequent co-application of NS1738 has little effect (Fig. 5A), a finding that is in agreement with previous studies conducted with another type I PAM (Young et al., 2008). In contrast, application of PNU-120596 to desensitized α7 nAChRs causes rapid reactivation, even in the continued presence of a desensitizing concentration of acetylcholine (Fig. 5B). Thus, persistent (non-desensitized) opening of α7 nAChRs requires the binding of an agonist to the orthosteric site and a type II PAM to its allosteric site. We would, therefore, expect recovery from desensitization to be prevented by the displacement of either acetylcholine or PNU-120596 from their respective binding sites. We have exploited this fact to examine whether type I and type II PAMs bind competitively.

### 3.3 Block of type II PAM-induced recovery from desensitization

**Fig. 6:**
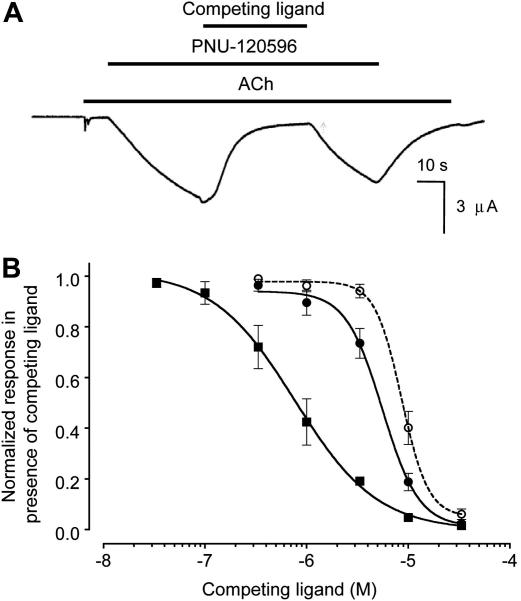
Block of recovery from desensitization by NS1738 and MLA. The ability of PNU-120596 to cause recovery from desensitization of α7 nAChRs is blocked by NS1738 and MLA. A) Representative trace showing activation of α7 nAChRs followed by rapid desensitization in response to acetylcholine (1 mM; lower horizontal line). Subsequent application of PNU-120596 (1 μM; middle horizontal line) allows recovery from desensitization. Recovery can be blocked by subsequent application of NS1738 (upper horizontal line). B) Dose-response curves showing the ability of NS1738 (filled circles) and MLA (filled squares) to block recovery from desensitization caused by co-application of acetylcholine (1 mM) and PNU-120596 (1 μM). Also shown (open circles) is the dose-response curve for NS1738 obtained when the experiment was performed in the presence of the same concentration of acetylcholine but with a higher concentration of PNU-120596 (3 μM). Data are means ± SEM of 3–7 independent experiments.

α7 nAChRs receptors were activated with a desensitizing concentration of acetylcholine (1 mM). After desensitization, receptors were then reactivated by the co-application of PNU-120596 (1 μM) in the continued presence of acetylcholine (Fig. 6). After recovery from desensitization had occurred, a range of concentrations of NS1738 were then applied in the continued presence of both acetylcholine and PNU-120596 (Fig. 6). NS1738 caused a dose-dependent inhibition of the recovery from desensitization (IC_50_ = 5.2 ± 0.4 μM, *n* = 3) (Fig. 6), a finding that can be explained by NS1738 causing displacement of PNU-120596 from its binding site. Similarly, a dose-depended inhibition was observed with the ‘orthosteric’ competitive antagonist MLA (IC_50_ = 0.8 ± 0.1 μM, *n* = 3; Fig. 6). Whereas the inhibition dose-response curve for MLA had a Hill coefficient close to unity (*n*_H_ = −1.1 ± 0.1, *n* = 3), the inhibition curve for NS1738 had a significantly steeper slope (*n*_H_ = −2.4 ± 0.4, *n* = 3). A plausible explanation for this difference is that MLA and NS1738 block recovery from desensitization by different mechanisms of action (displacement of acetylcholine from its orthosteric binding site by MLA and displacement of PNU-120596 from its allosteric binding site by NS1738). With the aim of testing this hypothesis, the ability of NS1738 to block recovery from desensitization was re-examined, but with a higher concentration of either PNU-120596 or acetylcholine. If NS1738 binds competitively at the PNU-120596 binding site, we would predict that higher concentrations of NS1738 to be required when the concentration of PNU-120596 was raised (a rightward shift of the dose-response curve would be expected), whereas raising the concentration of acetylcholine would not be expected to have an effect on inhibition by NS1738. When the experiment was repeated with a 3-fold higher concentration of PNU-120596 (3 μM, rather than 1 μM) but with the same concentration of acetylcholine (1 mM), a significant (*P* < 0.05, *n* = 3) 1.7-fold rightward shift in the NS1738 dose-response curve was observed (IC_50_ = 8.7 ± 0.9 μM, *n* = 4). In contrast, no significant shift was observed in the NS1738 dose-response curve when the experiment was repeated with a 10-fold higher concentration of acetylcholine (10 mM, rather than 1 mM) but with the same concentration of PNU-120596 (1 μM).

### 3.4 Computer docking simulations

**Fig. 7:**
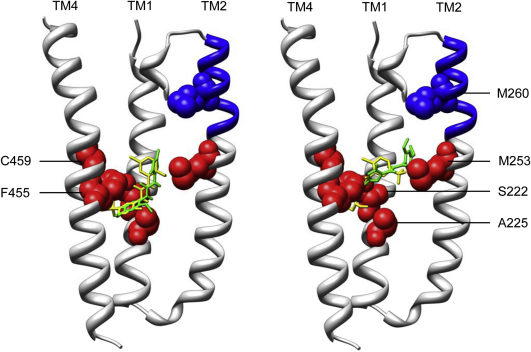
Computer docking simulation with a homology model of the α7 transmembrane domain. Computer docking simulations were performed with AutoDock 4 using a homology model of the α7 nAChR transmembrane domain (Cheng et al., 2007; Young et al., 2008). The backbone of the four transmembrane α helices (TM1-TM4) is shown in grey and the side chains of amino acids which, when mutated, had a significant effect on potentiation by both NS1738 and PNU-120596 (S222, A225, M253, F455 and C459) are shaded in red. The side chain of M260 (an amino acid that, when mutated, reduced receptor desensitization and had a selective effect on potentiation by NS1738) is shown in blue. The backbone region corresponding to the 10 amino acid ‘M2-M3 segment’, that has been described previously as having a selective effect on potentiation by NS1738 (Bertrand et al., 2008), is also shown in blue. The lowest energy (highest predicted binding affinity) docked positions of NS1738 and PNU-120596 are shown in yellow and green, respectively. Initially, a ‘blind docking’ approach was used in which no assumptions were made as to where within the transmembrane region the compounds might bind (left panel). Subsequently, more energetically favourable docked conformations were identified by conducting further simulations focussed upon the central region of the model (where the of the lowest energy docked conformations are located) using a modified homology model in which flexibility was permitted within the side chains of the five amino acid that have been identified as influencing potentiation of NS1738 and PNU-120596 (right panel). For clarity, the lower part of the TM3 α-helical domain has been omitted from the figure.

Computer docking simulations were performed with NS1738 using approaches that we have described previously for PNU-120596 (Young et al., 2008). Docking simulations employed a homology model of the transmembrane region of the human α7 nAChR (Cheng et al., 2006). As previously (Young et al., 2008), a ‘blind docking’ approach was used in which no assumptions were made about where within the transmembrane homology model NS1738 might bind. The most favourable (lowest energy) docked conformation of NS1738 was in a similar location to that identified previously for PNU-120596 (Fig. 7), providing support for the conclusion that NS1738 and PNU-120596 bind at a common or overlapping site. In addition, the predicted binding free energy (Δ*G*) for NS1738 (−7.95 kcal/mol; equivalent to a predicted binding affinity of 1.5 μM) to the homology model was similar to that reported previously for PNU-120596 (−7.98 kcal/mol; 1.41 μM) (Young et al., 2008). As found previously for PNU-120596, the lowest energy docked conformation of NS1738 was in very close proximity (within 6 Å) of the five amino acids that, when mutated, exert a significant effect on potentiation by NS1738 and PNU-120596 (S222, A225, M253, F455 and C459). Initially, blind docking simulations were performed by allowing flexibility of rotatable bonds within the ligand but with a rigid homology model. Subsequently, additional docking simulations were performed in which flexibility was allowed in the side chains of the five amino acids that had been identified as influencing potentiation by NS1738 and PNU-120596 (S222, A225, M253, F455 and C459) and by restricting the simulation to the central portion of the homology model. For both ligands, lower energy (higher predicted affinity) docked conformations were identified when flexibility of these five side chains was allowed (Fig. 7). The most favourable conformation of NS1738 identified using these docking conditions (Δ*G* = −12.07 kcal/mol; *K*_d_ = 1.42 nM) was of similar predicted binding energy to that reported previously for PNU-120596 in the semi-flexible homology model (Δ*G* = −11.65 kcal/mol; *K*_d_ = 2.87 nM).

## 4 Discussion

In a previous study that examined the modulation of α7 nAChRs by the type II positive allosteric modulator PNU-120596, it has been concluded that PNU-120596 acts via an allosteric intrasubunit transmembrane site (Young et al., 2008). Interestingly, a separate study has examined the ability of NS1738 to act as a positive allosteric modulator of α7 nAChRs and has demonstrated that potentiation by NS1738 is influenced selectively by a 10 amino acid ‘M2-M3 segment’ (Bertrand et al., 2008). This finding has, in turn, led to the suggestion that NS1738 may interact with the extracellular N-terminal domain (Bertrand et al., 2008). In particular, it was found that the extracellular segment between the second and third transmembrane domains (the M2–M3 segment) was important in modulating the allosteric effects of NS1738 but not of the type II PAM PNU-120596 (Bertrand et al., 2008). A reasonable interpretation of these previous studies might be that type I PAMs such as NS1738 and the type II PAM PNU-120596 interact with α7 nAChR at distinct binding sites. The experiments presented in this paper were, to a large extent, aimed at examining this possibility. The main conclusion that we have drawn from the experiments described herein is that NS1738 and PNU-120596 interact competitively at a common (mutually exclusive) transmembrane site. This conclusion is supported by studies of mutated α7 nAChRs (Fig. 3), experiments involving the co-application of NS1738 and PNU-120596 (Fig. 6) and by computer docking simulations (Fig. 7).

There is increasing evidence indicating that the α7 transmembrane region is the site of action for a range of allosteric modulators. In addition to evidence that this is the site of action of NS1738 and PNU-120596, it has been reported that ivermectin, a large macrocyclic lactone, also interacts with this region of the α7 nAChR (Collins and Millar, 2010; Sattelle et al., 2009). More recently, it has also been demonstrated that activation of α7 nAChRs by allosteric agonists can occur via this intrasubunit transmembrane site (Gill et al., 2011). There is, however, also strong evidence that allosteric modulators can interact with sites on the nAChR extracellular N-terminal domain. Galantamine, for example, acts as a nAChR potentiator (albeit much more weakly than PAMs such as NS1738 and PNU-120596) and has been demonstrated to bind at an extracellular site (Hansen and Taylor, 2007; Ludwig et al., 2010). In addition, there is evidence that morantel acts as an allosteric modulator of α3β2 nAChRs by binding to the extracellular domain (Seo et al., 2009). Interestingly, it has been proposed that morantel interacts with residues analogous to that of acetylcholine but at ‘noncanonical’ β-α subunit interface, rather than the conventional orthosteric α-β interface (Seo et al., 2009). Other recent studies have revealed the importance of non-acetylcholine-binding subunit interfaces in α4β2 nAChRs for modulation by Zn^2+^ (Moroni et al., 2008). The apparent diversity of modulatory sites on nAChRs is analogous to the situation in inhibitory Cys-loop receptors such as GABA- and glycine-gated ion channels, where modulatory sites have been identified at both the extracellular domain (Duncalfe and Dunn, 1996; Pritchett and Seeburg, 1991) and at transmembrane locations (Hosie and Wilkins, 2006; Mihic et al., 1997; Ye et al., 1998).

Despite the evidence presented here that NS1738 and PNU-120596 bind at a common transmembrane site, it is clear that there are some differences in the mechanism by which these two compounds interact with α7 nAChRs. Three of the amino acid mutations examined in the present study (S222M, A225D and M260L) differed significantly in the extent to which they altered levels of potentiation by NS1738 and PNU-120596 (Fig. 3). However, such findings are not unexpected, even if NS1738 and PNU-120596 interact competitively at a common or overlapping site. Such effects can be accounted for by differences in the chemical structure of ligands that interact with a common site. Indeed, we might have expected to find such differences in the effects of some mutations, given the dramatic difference in the extent to which these two compounds influence receptor desensitization.

Of the six mutations that were found to substantially reduce the potency of NS1738 as an allosteric modulator (S222M, A225D, M253L, M260L, F455A and C459Y), five (all except M260L) have significant and broadly similar effects on potentiation by NS1738 and PNU-120596. Interestingly, all of these five amino acids lie towards the bottom of the predicted intrasubunit transmembrane cavity (Young et al., 2008). The only mutation that we have identified that has a selective effect on NS1738 (M260L) is located towards the top of the TM2 helix (Figs. 4A and 7). Perhaps it is significant that this mutation also results in changes in receptor desensitization (Fig. 4B). Interestingly, M260L is located within the 10 amino acid region that was identified previously as being important in modulating the effects of NS1738 (Bertrand et al., 2008) and which was described in that study as the ‘short M2–M3 extracellular domain’. More recently, the same 10 amino acid region (described as being the ‘M2-M3 loop’) was found to be critical for potentiation by another type I PAM, genistein (Grønlien et al., 2010). However, although the 10 amino acid region identified in these two previous studies (amino acids AEIMPATSDS) lies beyond the region that is sometimes referred to as the TM2 (or M2) domain (Miller, 1989), examination of the high resolution (4 Å) structure of the *Torpedo* nAChR (Unwin, 2005) reveals that this 10 amino acid region lies entirely within the TM2 α-helical region, albeit extending partly above the lipid bilayer. As is illustrated in the α7 homology model (which was derived from the *Torpedo* nAChR structure), M260 lies within the extended α-helical region, close to the top of the transmembrane region (Figs. 4A and 7). Interestingly, early models of the helical transmembrane region of the *Torpedo* nAChR, generated prior to its detailed three-dimensional structural determination and based on known biophysical properties of α helices, suggested that most of this 10 amino acid region corresponded to an α-helical structure located within the polar head region of the lipid bilayer (Popot and Changeux, 1984). However, it has recently been pointed out that there are differences between the TM2 region of the *Torpedo* nAChR structure and the TM2 region from the higher resolution structures such as the invertebrate glutamate-gated chloride channel (GluCl) and bacterial ion channels (Corringer et al., 2010; Hibbs and Gouaux, 2011). A homology model of the α7 nAChR derived from GluCl would place only the first half of the 10 amino acid region within the TM2 α-helical region of and would locate M260 at the start of the region linking TM2 and TM3.

Computer docking simulations (Fig. 7) support the idea that both NS1738 and PNU-120596 bind at a site close to the five amino acids that have been identified as exerting a significant effect on allosteric modulation by both PAMs. In contrast, M260 is located further away (approximately 9 Å). It is possible that the selective effect of M260 is primarily a consequence of its effects on receptor biophysical properties such as desensitization, rather than a more direct effect at the PAM binding site.

The difference in Hill slopes of inhibition curves for MLA and NS1738 observed in our co-application experiments (Fig. 6) are consistent with the possibility that the effects of these compounds in blocking recovery from desensitization may be due to a different mechanism of action. Whereas MLA would be expected to cause displacement of acetylcholine from its extracellular orthosteric binding site, it is plausible that NS1738 acts by causing displacement of PNU-120596 from its allosteric transmembrane site. This conclusion is supported by our finding that changing the concentration of PNU-120596 has a significant effect on the inhibition curve for NS1738, whereas changing the concentration of acetylcholine does not (Fig. 6B). This is the result that would be expected if NS1738 binds competitively with PNU-120596 but binds independently of acetylcholine. It could be argued that NS1738 is acting as a non-competitive antagonist with respect to PNU-120596, by binding to a third site on the receptor (i.e. distinct from both the orthosteric acetylcholine binding site and the allosteric PNU-120596 binding site). This seems unlikely for several reasons. It would require NS1738 to be exerting a powerful non-competitive antagonist effect that can surmount the potentiating effect of PNU-120596. There is no evidence that NS1738 has any direct antagonist action, indeed it acts as a potent potentiator when co-applied with acetylcholine. The chemical similarity of NS1738 and PNU-120596 (Fig. 1), both of which are aromatic-linked urea compounds, would seem to make the likelihood of a common binding site plausible and is supported by computer docking simulations (Fig. 7). Taken together, the data presented in this study support the conclusion that binding of NS1738 and PNU-120596 to α7 nAChR is mutually exclusive, just as the binding of agonists and competitive agonists (such as acetylcholine and MLA) is at the orthosteric ligand binding site.

## 5 Conclusion

The simplest explanation for these findings is that acetylcholine and MLA bind competitively at the orthosteric binding site (as is well established) and that NS1738 and PNU-120596 bind competitively at a common or overlapping allosteric binding site. This explanation for the actions of NS1738 and PNU-120596 is supported by studies examining the effect of point mutations located at the postulated transmembrane binding site. This conclusion is also supported by computer docking simulations that predict that the most favourable (lowest energy) binding site for NS1738 and PNU-120596 is at a common transmembrane site, located in very close proximity to mutations known to affect the ability of NS1738 and PNU-120596 to act as positive allosteric modulators.

## Figures and Tables

**Table 1:** Influence of α7 point mutations upon potentiation by NS1738 and PNU-120596.

Subunit/Mutation	Location	Fold potentiation by NS1738	Fold potentiation by PNU-120596
Wild type		39.5 ± 2.7	36.7 ± 4.1
G211V	TM1	45.9 ± 4.3	35.0 ± 2.2
S222M	TM1	6.3 ± 0.5***	25.7 ± 1.4*
A225D	TM1	9.1 ± 0.7***	3.2 ± 0.3***
L230Y	TM1	32.2 ± 2.2	40.1 ± 5.3
M253L	TM2	1.9 ± 0.1***	4.1 ± 0.8***
M260L	TM2	4.0 ± 0.3***	28.5 ± 2.0
Q272V	TM3	28.3 ± 5.3	38.4 ± 0.2
S276V	TM3	40.7 ± 2.2	40.3 ± 2.7
F455A	TM4	11.1 ± 1.1***	24.7 ± 3.8*
T456V	TM4	41.1 ± 1.3	38.3 ± 1.7
C459Y	TM4	14.3 ± 3.3***	15.4 ± 1.3***

Potentiation by a maximal concentration (10 μM) of NS1738 reflects the increase in agonist response with an EC_20_ concentration of acetylcholine and are compared to data obtained with a maximal concentration (3 μM) of PNU-120596 (Young et al., 2008). As reported previously (Young et al., 2008), in most cases mutations had a minimal effect on acetylcholine potency (the EC_20_ concentrations of acetylcholine used in these experiments were 20 μM for M260L, 60 μM for Q272V and 50 μM for all other mutations and for wild type α7 nAChRs). Data are means of 3–10 independent experiments, ±SEM. Statistical significance, determined by ANOVA and Tukey’s test (NS1738 data *F*_(11,65)_ = 64.6; PNU-120596 data *F*_(11,42)_ = 25.7), *P < 0.05; ***P < 0.001.
